# Useulness of B Natriuretic Peptides and Procalcitonin in Emergency Medicine

**DOI:** 10.4137/bmi.s499

**Published:** 2008-03-27

**Authors:** S. Delerme, C. Chenevier-Gobeaux, B. Doumenc, P. Ray

**Affiliations:** 1 Department of Emergency Medicine and Surgery, Centre Hospitalo-Universitaire Pitié-Salpêtrière, Assistance-Publique Hôpitaux de Paris (AP-HP), 47-83 boulevard de l’hôpital, 75013 Paris, Université Pierre et Marie Curie Paris 6, France; 2 Department of Biochemistry A, Hôpital Cochin, Assistance Publique-Hôpitaux de Paris (AP-HP), 27 rue du Faubourg Saint-Jacques, 75679 Paris Cedex 14, France and; 3 Department of Emergency Medicine, Centre Hospitalo-Universitaire de Bicetre, Assistance-Publique Hôpitaux de Paris (AP-HP), 94270 Kremlin-Bicetre, Université Paris Sud 11, France

**Keywords:** dyspnea, NT-proBNP, BNP, heart failure, emergency department, septic shock, community-acquired pneumonia

## Abstract

Congestive heart failure (CHF) is the main cause of acute dyspnea in patients presented to an emergency department (ED), and it is associated with high morbidity and mortality. B-type natriuretic peptide (BNP) is a polypeptide, released by ventricular myocytes directly proportional to wall tension, for lowering renin-angiotensin-aldosterone activation. For diagnosing CHF, both BNP and the biologically inactive NT-proBNP have similar accuracy. Threshold values are higher in elderly population, and in patients with renal dysfunction. They might have also a prognostic value. Studies demonstrated that the use of BNP or NT-proBNP in dyspneic patients early in the ED reduced the time to discharge, total treatment cost. BNP and NT-proBNP should be available in every ED 24 hours a day, because literature strongly suggests the beneficial impact of an early appropriate diagnosis and treatment in dyspneic patients.

Etiologic diagnosis of febrile patients who present to an ED is complex and sometimes difficult. However, new evidence showed that there are interventions (including early appropriate antibiotics), which could reduce mortality rate in patients with sepsis. For diagnosing sepsis, procalcitonin (PCT) is more accurate than C-reactive protein. Thus, because of its excellent specificity and positive predictive value, an elevated PCT concentration (higher than 0.5 ng/mL) indicates ongoing and potentially severe systemic infection, which needs early antibiotics (e.g. meningitis). In lower respiratory tract infections, CAP or COPD exacerbation, PCT guidance reduced total antibiotic exposure and/or antibiotic treatment duration.

## Introduction

**Figure 1:**
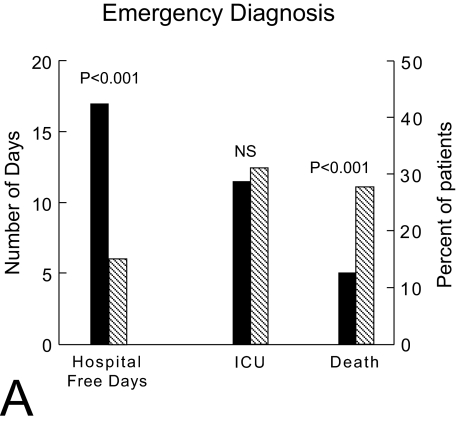
Effects of an appropriate (black bars) or inappropriate (white bars) initial diagnosis in the emergency department on prognosis (used with permission from Ray et al. 2006a).

The number of emergency department (ED) admissions continues to climb, with a limited number of departments available to handle the volume (McCaig and Burt, 2004). The rapid and accurate diagnosis of the most severe patients is a routine challenge for emergency physicians. Unfortunately in patients suspect of sepsis, symptoms and clinical signs, and current routine laboratory tests lack both sensitivity and specificity in correctly identifying which patients have sepsis and need antibiotics (Hausfater et al. 2002). Likewise, the signs and symptoms of congestive heart failure (CHF) are frequently nonspecific, highly variable especially in older adults or patients with pre-existing respiratory diseases (Rihal et al. 1995; Lien et al. 2002; Mueller et al. 2005a). Indeed, CHF can present with wheezing and mimic acute asthma, so-called cardiac asthma (Jorge et al. 2007). However, new evidences show that early appropriate interventions can reduce mortality rate in patients with sepsis and improve outcome in acute dyspnea (Rivers et al. 2001; Mueller et al. 2004). Rivers et al. demonstrated a 16% decrease in 28-day mortality rate by simply implementing a protocol mandating aggressive ED resuscitation during the first 6 hrs of a patient’s ED visit. Likewise, studies suggested that an early appropriate diagnosis and therapy is associated with a decreased mortality from acute dyspnea/CHF (Fig. 1) (Ray et al. 2006a). Thus, available biological tools—namely biomarkers—that could provide a rapid diagnosis of bacterial infection in case of fever/sepsis and rapid diagnosis of CHF in case of dyspnea could be very useful (Maisel et al. 2002; Mueller et al. 2004; Jones et al. 2007). Furthermore, their ability to accurately assess a patient’s disease severity and mortality risk at ED presentation could be also important (Meisner, 2005). The ideal biomarker would be readily available, technically easy to perform with a quick turn-around time, inexpensive, highly specific, very sensitive, and preferably highly correlated in quantitative terms with disease severity. Such a test would provide early diagnostic accuracy, prognostic information, and indicate responsiveness to treatment interventions. Regrettably no such ideal biomarker exists at present. However, the purpose of this review is to indicate recent developments in biomarkers of sepsis and heart failure to evaluate their impact on clinical use in the emergency setting. Thus, we will focus on the rationale, and use of B natriuretic peptides (NP) in CHF and procalcitonin (PCT) in sepsis in adults.

## Role of the Natriuretic Peptides in Heart Failure

### Physiologic secretion of natriuretic peptides (NPs)

An important pathophysiologic mechanism in cardiovascular disease is the imbalance between the vasoconstrictive/antinatriuretic action of some neuroendocrine factors (de Bold et al. 1981; Clerico et al. 2006). Cardiac endocrine function is an essential component of the homeostatic regulation network, including the renin-angiotensin-aldosterone system, vasopressin, endothelins, and sympathetic nervous system, and the counter-regulatory vasodilatory/natriuretic response, mainly represented by NP (Yasue et al. 1994). As cardiac performance decreases, all neurohormonal systems are progressively stimulated in an attempt to sustain cardiac output and circulatory homeostasis. However, the activation of neurohumoral mechanisms may worsen the hemodynamics, and have direct adverse effects on myocardial function, and stimulate the NPs (Levin et al. 1998).

**Figure 2:**
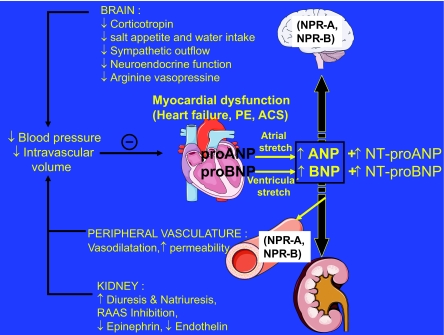
Physiologic actions of BNP.

The NP have several physiologic actions (Fig. 2), the most important being *(a)* vasodilation; *(b)* promotion of natriuresis and diuresis; *(c)* inhibition of the sympathetic nervous system and of the renin-angiotensin-aldosterone system, endothelins, cytokines, and vasopressin; *(d)* inhibition of the pathophysiologic mechanisms responsible for ventricular and vascular hypertrophy and remodeling; and *(e)* beneficial effects on endothelial dysfunction secondary to the atherosclerotic process, including blunting of shear stress and regulation of coagulation and fibrinolysis, as well as inhibition of platelet activation (Levin et al. 1998; Maisel, 2003).

The NP family includes atrial natriuretic peptide (ANP), B-type natriuretic peptide (BNP) and its related peptide, whereas C-type natriuretic peptide (CNP) and urodilatin (DNP) are predominantly secreted by noncardiac tissues (endothelium and kidney, respectively) (Ruskoaho, 2003).

**Figure 3:**
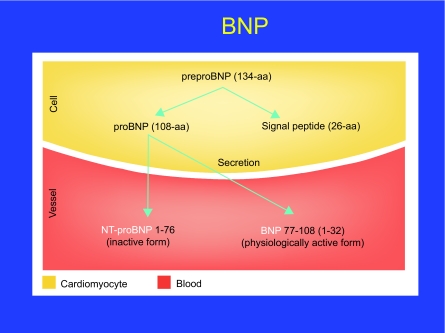


BNP derives from the precursor pre-proBNP, containing 134 amino acids and including a signal peptide of 26 amino acids. ProBNP, produced by cleavage of the signal peptide, is further split into BNP, which is considered to be the biologically active hormone, and an inactive N-amino terminal fragment, NT-proBNP (Fig. 3). NT-proBNP is used in a commercial analytical method and refers to measurement of N-terminal 1–76 fragment. BNP is a 32-aa polypeptide containing a 17-aa ring structure common to all NP (Maisel et al. 2002). BNP gene expression is a feature of both atrial and ventricular myocytes. In the healthy heart, BNP gene expression occurs mainly in the atria. However, ventricular BNP gene expression is up-regulated in diseases that affect the ventricles, such as CHF, which suggests that it may be a more specific indicator of ventricular disorders than other NP. It is a well-established fact that atrial myocytes contain secretory granules for peptide storage, which led to the primary hypothesis about the endocrine heart (Levin et al. 1998). Importantly, atrial granules store both intact proBNP and cleaved products, i.e. bioactive BNP-32. In contrast, ventricular myocytes in the healthy heart do not seem to produce these granules, and do not contain proBNP-derived peptides. The nucleic acid sequence of the BNP gene contains the destabilizing sequence “TATT-TAT,” which suggests that turnover of BNP messenger RNA is high and that BNP is synthesized in bursts. This release appears to be directly proportional to ventricular volume expansion and pressure overload (Levin et al. 1998).

The clearance of the two peptides is different (Anand-Srivastava, 2005). BNP is cleared by several mechanisms, including the kidneys, specific clearance receptor-mediated degradation (natruretic peptide receptor type C, NPR-C) and enzymatic degradation, especially neutral endopeptidase. In contrast, NT-proBNP seems to be removed exclusively by the kidneys (Austin et al. 2006). These differences in clearances are responsible for the fact that BNP has a lower absolute plasma concentration and a lower half-life. However, Kroll et al. recently re-calculated the NT-proBNP half-life and found that it was closer to that of BNP (25 minutes) (Kroll et al. 2007).

### Factors related to natriuretic peptide secretions

NP production are greatly increased in diseases characterized by an expanded fluid volume, including renal failure, liver cirrhosis, and CHF (Clerico et al. 1998). The circulating concentrations of NPs are also modified by several physiologic factors, such as circadian variations, sodium intake, and drugs (including corticosteroids, diuretics, angiotensin-converting enzyme inhibitors, and adrenergic agonists and antagonists). However, the main variations in circulating concentrations of NPs in healthy adults are related to weight, aging and gender. In particular, the BNP concentration is about one third higher in women than in men at age <50 years (Emdin et al. 2003). The higher NPs values in women could be explained by the physiologic stimulation of female sex steroid hormones. The increases in NPs with aging may be also attributable to physiologic cardiac hypertrophy and kidney’s senescence (Loke et al. 2003). Moreover, the increase in NPs with aging may be attributable to a decrease in their clearance rate (Ray et al. 2006a). When adjusted for relevant covariates, compared with normal counterparts, overweight and obese patients with acute CHF have lower circulating NT-proBNP and BNP levels, suggesting a BMI (body mass index)-related defect in natriuretic peptide secretion (Das et al. 2005). However, a recent study suggested that the association between BMI and BNP and NT-proBNP could be mediated by lean mass rather than fat mass (Das et al. 2005), and these results do not support the hypothesis that the lower BNP levels seen in obesity are driven by enhanced BNP clearance mediated via NPR-C. In fact, studies on effect of obesity on natriuretic peptides are controversial (Hermann-Arnhof et al. 2005).

There is a strong inter-relationship between heart failure (therefore BNP and NT-proBNP concentrations) and renal function. Therefore, the central questions are i) how to interpret BNP and NT-proBNP concentrations in patients with renal impairment; ii) is BNP superior to NT-proBNP in case of renal dysfunction? Januzzi et al. found that NT-proBNP and eGFR (glomerular filtration rate) were inversely and independently related (Anwaruddin et al. 2006). McCullough et al. determined that BNP concentrations and cut-off points were influenced by renal function, particularly in those with an eGFR (glomerular filtration rate) less than 60 mL/min/1.73 m^2^ (McCullough et al. 2003). Chenevier-Gobeaux et al. showed that (i) both NT-proBNP and BNP values were inversely correlated to eGFR, and (ii) the lower was the eGFR, the higher were the cut-off values of NT-proBNP and BNP (Chenevier-Gobeaux et al. 2005). Conversely, Ray et al. demonstrated that BNP was more accurate than NT-proBNP in elderly patients, because of a decrease in creatinine clearance (Ray et al. 2005).

### Their diagnostic role in dyspnea

BNP is an independent predictor of high left ventricular end-diastolic pressure, or high capillary pulmonary artery pressure (Maeda et al. 1998). Both correlate to the New York Heart Association classification, the severity of the heart failure and inversely correlate to left ventricular ejection fraction (Davis et al. 1994; Januzzi et al. 2005). Cardiologic studies showed that BNP and NT-proBNP can reliably predict the presence or absence of left ventricular dysfunction on echocar-diogram (Lubien et al. 2002).

The potential clinical usefulness of BNP and NT-proBNP for differential diagnosis of dyspnea and for prognostic stratification of patients with CHF has been confirmed in the last 5 years. Thus, the Task Force of the European Society of Cardiology recommended that a NPs assay should be included in the first step of the algorithm for the diagnosis of HF as electrocardiography (ECG) and chest x-rays (Swedberg et al. 2005).

Acute dyspnea is the key symptom of most respiratory diseases, with high related morbidity and mortality (Ray et al. 2006a). Unfortunately, emergency physicians’ accuracy to diagnose CHF is less than 60%–70% (McCullough et al. 2003; Ray et al. 2006a). In the EPIDASA study (Ray et al. 2006a), 514 patients older than 65 years with acute dyspnea were included. The in-hospital mortality was 16%, with a higher mortality (21%) in the 219 (42%) patients with CHF. An inappropriate emergency treatment occurred in 162 (32%) patients, and led to a higher mortality (25% versus 11%; p < 0.001), highlighting the importance of an early correct diagnosis and treatment in the ED (Fig. 1).

Numerous studies evaluated and validated both NPs to diagnose CHF in acute dyspnea in adults patients (Davis et al. 1994; Cabanes et al. 2001; Dao et al. 2001; Logeart et al. 2002; Maisel et al. 2002; Lainchbury et al. 2003; Bayes-Genis et al. 2004; Ray et al. 2004; Alibay et al. 2005; Chenevier-Gobeaux et al. 2005; Januzzi et al. 2005; Mueller et al. 2005b; Ray et al. 2005; Berdague et al. 2006). The largest studies were performed by Maisel et al. with BNP (Maisel et al. 2002) and Januzzi et al. (Januzzi et al. 2005) with NT-proBNP. All these studies used the same methodology: there were prospective studies of consecutive patients who came to EDs with acute dyspnea and whose BNP/NT-proBNP was measured—blindly—at admission (Maisel et al. 2002). The clinical diagnosis of CHF was adjudicated by independent experts, who were blinded to the results of the BNP/NT-proBNP assay, according to the summary chart and echocardiographic findings; usual exclusion criteria were severe renal insufficiency, patients whose dyspnea was clearly not secondary to CHF (chest trauma), and dyspnea secondary to severe coronary ischemia.

In the Maisel’s study, BNP levels by themselves were more accurate than any historical or physical findings or laboratory values in identifying CHF as the cause of dyspnea. The diagnostic accuracy of BNP at a cutoff of 100 pg/ml was 83.4% with an area under the receiver-operating-characteristic curve (AUC) of 0.91; and BNP was more accurate (83%) than the Framingham criteria (73%), a commonly used set of criteria for diagnosing CHF. In fact, the analysis of their ROC curve reveals that the best threshold value which should have been defined by the highest accuracy of 84% was close to 150 pg/ml (with a lower sensitivity of 85%).

The PRIDE (ProBNP Investigation of Dyspnea in the Emergency Department) study included 600 patients (mean age of 67 years) (Januzzi et al. 2005). A NT-proBNP level <300 pg/ml was optimal for ruling out CHF, with a negative predictive value of 99%. Again, NT-proBNP testing alone was superior to clinical judgment alone for diagnosing acute CHF (p = 0.006). Using an age categorization of <50 years (n = 144) and ≥50 years (n = 455), they determined that—for ruling in acute CHF—the optimal cut off were 450 and 900 pg/ml with areas under the curve of 0.98 and 0.93, respectively. However, the major criticism was the use of 2 different cut-off depending of the age of the population studied.

### Specific considerations in elderly

To our knowledge, only 3 studies specifically have evaluated BNP and NT-proBNP in elderly patients (Ray et al. 2004; Arques et al. 2005; Berdague et al. 2006). Ray et al. included 314 patients with acute dyspnea. They demonstrated that increased BNP was the strongest independent predictor of a final diagnosis of CHF (odds ratio (OR) 24.4, p < 0.001), and the accuracy of BNP-assisted diagnosis was higher than that of the emergency physician (0.84 versus 0.77, p < 0.05). They also showed that, whatever the empirical probability for CHF (from absent to very likely), estimated by the emergency physician, an elevated level of BNP higher than 250 pg/ml was an accurate marker of CHF (positive likelihood ratio from 5.9 to 8.8, respectively). Berdague et al. assessed the usefulness of NT-proBNP assay for the diagnosis of CHF in 256 elderly patients (mean age 81 years). The diagnoses made in the ED were incorrect or uncertain in 45% of cases. NT-proBNP >2000 pg/ml was the most powerful independent marker of cardiac dyspnea (OR 13.6, p < 0.001). Lastly, Arques et al. demonstrated that BNP had an AUC of 0.875 for predicting CHF with preserved LV systolic function (Arques et al. 2005). Overall in the elderly, BNP and NT-proBNP are both accurate to diagnose CHF. However, threshold values are higher.

**Figure 4:**
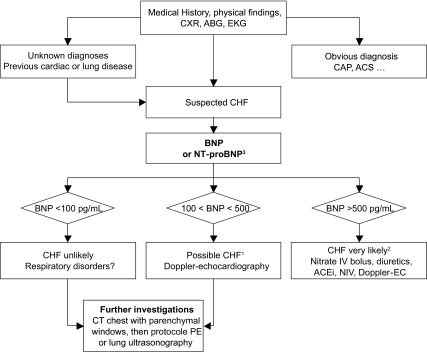
Diagnostic strategy based on B natriuretic peptide levels in elderly patients admitted for ARF in the emergency department. ^1^In the grey zone (BNP between 100 and 500 pg/mL) which represents less than a quarter of patients, further investigations are needed, and ER physicians should consider massive PE, severe exacerbation of COPD or severe pneumonia as possible diagnoses… ^2^Emergency physicians should be keep in mind that half of elderly patients with ARF has more than one, i.e. a BNP greater than 500 pg/ml strongly suggests CHF, but other diagnosis could have precipitated CHF. ^3^For NT-proBNP, the cut-off values are 500 and 2,000 pg/mL. **Abbreviation:** CXR: chest x- ray; EKG: Electrocardiogram; ABG: arterial blood gas analysis; CHF: congestive heart failure; ACS: acute coronary syndrome, CT: computed tomography; IV: intra-venous; NIV: non invasive ventilation including continuous positive airway pressure; ACEi: angiotensin converting enzyme inhibitor; EC: echocardiography.

Roughly, CHF appears to be highly unlikely below a BNP plasma concentration of 100 pg/ml, or a NT-proBNP below 500 pg/mL, and CHF appears to be likely when plasma concentration of BNP is higher than 500 pg/ml, or NT-proBNP is greater 2000 pg/mL (Fig. 4). Between these situations (so-called “the grey zone”), further investigation are needed (such as echocardiography, CT chest…, since many causes might be suspected (including atrial fibrillation without CHF, pulmonary embolism, exacerbation of COPD (chronic obstructive respiratory disease), severe pneumonia, severe sepsis and CHF) (Ray et al. 2004; Knudsen et al. 2005; Maeder et al. 2006).

### Comparison between BNP and NT-proBNP

In most of the studies, BNP and NT-proBNP were well correlated (Alibay et al. 2004; Clerico et al. 2006; Lainchbury et al. 2003; Ray et al. 2005). Whether BNP has a better accuracy than NT-proBNP has been a matter of debate. Ray et al. included 202 patients (mean age of 80 years) in whom 88 (44%) had CHF. For diagnosing CHF, the AUC for NT-proBNP was lower than that of BNP (0.80 vs. 0.85, p < 0.05). Using logistic regression, only the diagnosis of CHF predicted the elevation of BNP greater than 250 pg/mL (OR = 27.7, p < 0.001). Conversely, beside the diagnosis of CHF (OR = 11.7, p < 0.001), a creatinine clearance of less than 60 mL/min (OR = 2.7; p < 0.05) independently predicted the elevation of NT-proBNP greater than 1,500 pg/mL. It might be the renal dysfunction partly explained the lower accuracy of NT-proBNP observed, because in contrast to BNP, renal excretion is a major route of elimination of NT-proBNP (Ray et al. 2005).

However, the other studies compared diagnostic accuracy of BNP and NT-proBNP, even in an elderly population, and did not confirm this previous findings. In a subgroup (n = 75) analysis of their study, Berdague et al. demonstrated that AUC was 0.86 for BNP compared with 0.88 for NT-proBNP (p = 0.6) (Berdague et al. 2006). In 160 patients over 75 years of age, Alibay et al. demonstrated that the diagnostic value was similar for BNP and NT-proBNP (AUC of 0.82 vs. 0.84, respectively) (Alibay et al. 2004). Mueller et al. demonstrated that AUC for BNP and NT-proBNP in patients with dyspnoea did not differ significantly (AUC of 0.916 vs. 0.903) (Mueller T et al. 2005). Chenevier-Gobeaux et al. found that NT-proBNP and BNP concentrations increased in a similar way when eGFR decreased in 381 patients (mean age of 79 years) with dyspnoea (Chenevier-Gobeaux et al. 2005). NT-proBNP (and BNP) cut-off points rose as a function of eGFR categories: from 1360 (and 290) pg/ml in patients with eGFR 60–89 ml/min/1.73 m^2^, to 6550 (and 515) pg/ml in patients with eGFR 15–29 ml/min/1.73 m^2^.

Overall BNP and NT-proBNP might be equally useful as an aid in the diagnosis of CHF in patients presenting to the ED with shortness of breath.

### Do NT-proBNP and BNP improve outcome?

There is now evidence that early measurement of BNP or NT-proBNP following admission to the ED could improve the outcome of dyspneic patients. Mueller et al. conducted a prospective, randomized, controlled study of 452 patients who presented to the ED with acute dyspnea: 225 patients were randomly assigned to a diagnostic strategy involving the measurement of BNP levels with the use of a rapid bedside assay, and 227 were assessed in a standard manner (Fig. 4). The use of BNP levels reduced the need for hospitalization (75% vs 85%). Patients in the BNP group spent less time in the hospital (8 *vs.* 11 days), and their care cost was less ($5410 *vs.* $7264) than those whose physicians used conventional assessment. The respective 30-day mortality rates were unchanged. However, a significant reduction in 30-day mortality was observed (9% in the BNP group vs. 17% in the control group; p = 0.039) in the sub-group of patients older than 70 years (Mueller et al. 2004). Although echocardiography and pulmonary function testing were strongly recommended for patients, the percentage actually having the tests was not reported (Mueller et al. 2004).

Moe et al. also demonstrated that NT-proBNP testing improves the management of patients presenting with dyspnea (Moe et al. 2007). Knowledge of NT-proBNP results reduced the number of patients rehospitalized over 60 days by 35% (51% to 33%), and direct medical costs of all ED visits, hospitalizations, and subsequent outpatient services (US $6129 to US $5180 per patient) over 60 days from enrollment. However, knowledge of NT-proBNP results did not result in any major improvement in clinical outcomes to 60 days, including lack of significant differences in the initial rate of hospitalization, hospital length of stay, or mortality rates.

### Other potential uses of natriuretic peptides assessment in acute dyspnea

Studies suggest that high BNP or NT-proBNP level at admission could be of prognostic importance (Harrison et al. 2002; Logeart et al. 2004; Bayes-Genis et al. 2005; Gegenhuber et al. 2006; Januzzi et al. 2006).

**Figure 5:**
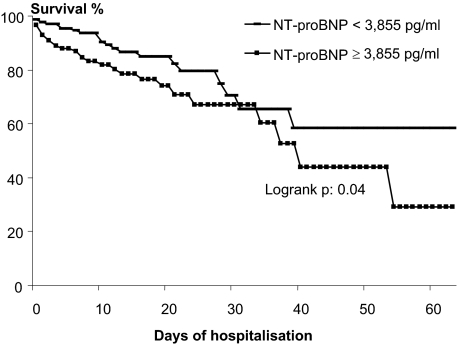
Kaplan-Meier curves showing survival according to NT-proBNP (from Chenevier-Gobeaux with permission (Chenevier-Gobeaux et al. 2007)).

Januzzi et al. demonstrated that NT-proBNP at the time of presentation was not only diagnostically useful, but also strongly predicted likelihood for short-term mortality in subjects with CHF, with a more than five-fold increase in risk for death by 76 days among those with marked elevation in NT-proBNP concentrations (Januzzi et al. 2006). Gegenhuber et al. followed up 251 consecutive patients (mean age of 72 years) admitted for shortness of breath (Gegenhuber et al. 2006). Mortality was higher in patients with baseline BNP and NT-proBNP concentrations above cut off levels. In a multivariate analysis of the EPIDASA study, elevated NT-proBNP or BNP (odds ratio 2.06) was also predictive of death (Ray et al. 2006a). However, other studies suggest that BNP or NT-proBNP values at predischarge rather than at admission, could be more accurate for predicting long-term outcome in patients admitted for acute CHF. Furthermore, changes in BNP levels during acute cares is also of interest (Logeart et al. 2004). Logeart et al. included 114 patients with severely decompensated CHF. They found that mean BNP levels were 1,015 pg/ml at admission, 881 ± 615 ng/l at 24 h, 638 pg/ml at 48 h, and 457 pg/ml predischarge. Recently, Chenevier-Gobeaux et al. reported that NT-proBNP higher than 3,855 pg/ml at admission was associated with higher in-hospital mortality in 324 patients aged 75 years and over admitted for dyspnea (17.9% vs. 9.7%, Fig. 5) (Chenevier-Gobeaux et al. 2007).

### Prognostic value in pulmonary embolism

Like in acute coronary syndrome (Morrow et al. 2005), studies have suggested that BNP or NT-pro-BNP (as cardiac Troponin) were accurate in risk stratification for PE in a middle-aged population and had a high positive predictive value for in-hospital death (Binder et al. 2005; Pieralli et al. 2006; Logeart et al. 2007). However, a study evaluated the usefulness of BNP in risk stratification of PE in 51 patients older than 65 years (79 ± 9 years) patients. Although, the median BNP level was significantly higher in the group of complicated PE (274 pg/mL vs. 78 pg/mL, p < 0.05), BNP was not a solid test to identify patients with a risk of complicated PE in elderly patients (AUC of only 0.72, p < 0.05) (Ray et al. 2006b). Thus, in clinical practice the measurement of BNP or NT-proBNP level for each PE admitted to an ED cannot be recommended.

### NP in practice

BNP and NT-proBNP have similar accuracy in acute dyspnea to diagnose CHF, even in elderly or “renal” patients. They probably have a prognostic value. Use of BNP or NT-proBNP should be promoted in the ED to assist in triage (CHF or not). Thus, their measurements should be strongly promoted in ED, especially when the cause of dyspnea in uncertain in order to improve costs and morbidity.

## Role of the PCT in Case of Fever or Suspicion of Sepsis

### Introduction about PCT and CRP

Among several markers of inflammation and sepsis, PCT and C-reactive protein (CRP) markers are being studied to investigate their accuracy for the diagnosis of bacterial infections. PCT is the prehormone of calcitonin, which is normally secreted by the C cells of the thyroid in response to hypercalcemia; under these normal conditions, negligible serum PCT concentrations are detected (Muller et al. 2000). The mechanism proposed for PCT production after inflammation and its role are still not completely known. It is believed that PCT is produced by the liver and other organs (adipocytes, lungs, muscles cells) (Spapen et al. 2006) and peripheral blood mononuclear cells, modulated by lipopolysaccharides and sepsis-related cytokines (Tumor Necrosis Factor α, IL-2, IL-6). PCT secretion begins within 4 h after stimulation and peaks at 8 h, clearing when the insult is under control (Harbarth et al. 2001). PCT levels are therefore very low (<0.05 ng/ml) in healthy humans. However, during severe infections (especially bacterial) with systemic manifestations PCT levels increase to over 100 ng/ml. Remarkably, the large amounts of PCT produced during infections do not lead to an increase in plasma calcitonin levels or activity. In contrast to the short half-life of calcitonin (10 min), PCT has a long half-life of approximately 22–35 h in serum. During severe systemic infections PCT is probably produced by extrathyroid tissues. Thus, patients who have previously undergone total thyroidectomy can still produce high levels of PCT during a severe infectious episode. PCT is stable in samples, the assay is relatively easy to perform, with a moderate cost (~$15), and the result is available within 1/2 h.

CRP is an acute-phase reactant, and CRP level measurements are frequently used to aid in the diagnosis of bacterial infections. CRP is synthesized by the liver, mainly in response to IL-6, which is produced not only during infection but also in many types of inflammation (Clyne and Olshaker, 1999). It binds to polysaccharides in pathogens, activating the classical complement pathway. CRP secretion starts within 6 h after stimulation, peaking only after 36 h. The assay for determing CRP levels is easy to perform, often automated, quick and has a lower cost (~$5).

### Why do we need a biomarker of sepsis in our EDs?

Etiologic diagnosis of febrile patients who present to an ED is complex and sometimes difficult. Physicians have to identify and often rapidly treat patients with systemic bacterial infection. However, the empirical use of broad-spectrum antibiotics in patients without infection is potentially harmful, facilitating colonization and superinfection with multidrug resistant bacteria. Most microbiological test (blood cultures) results are not available for 24 h. Clinical signs of SIRS (systemic inflammation response syndrome) including changes in body temperature, tachycardia, and leukocytosis are neither sensitive enough nor specific enough for the diagnosis of sepsis and can often be misleading. Although CRP level is a very sensitive marker of inflammation, it lacks specificity and so has limited utility in the ED. Trauma, burns, pancreatitis, major surgery, and many other conditions may elicit clinical signs of SIRS in the absence of microbial infection. There is no gold standard for diagnosing sepsis, as cultures may be negative especially in cases of antibiotic pre-treatment or inadequate sampling. Indeed, more than 30% of infected patients remain without clear documentation of microbial infection. Among 2,154 septic shock patients only 50% of patients received effective antimicrobial therapy within 6 hrs of documented hypotension. The multivariate analysis revealed that time to initiation of effective antimicrobial therapy was the single strongest predictor of outcome. Furthermore, each hour of delay in antimicrobial administration over the ensuing 6 hrs was associated with an average decrease in survival of 7.6% (Kumar et al. 2006). Although its specificity and sensitivity are poor, serum lactate is used for diagnosing septic shock (Levy et al. 2003). Lactate could be a biologically plausible candidate for risk-stratification biomarkers in ED patients with infection (Shapiro et al. 2005).

However, among the potentially useful markers of sepsis, PCT has been suggested to be the most promising, although results are variable depending on the severity of illness and patient population studied.

### Overall results in ICU and internal medicine

Three recent meta-analysis reported conflict results in ICU or in ED. Jones et al. found the diagnostic performance of the PCT test for identifying bacteremia in ED patients to be moderate, with an AUC of 0.84 and a sensitivity and specificity of 76% and 70%, respectively (Jones et al. 2007). Uzzan et al. found that global odds ratios for diagnosis of infection complicated by systemic inflammation were 15.7 using PCT and 5.4 using CRP. The AUC for PCT was better than for CRP (0.78 vs. 0.71, p = 0.02) (Uzzan et al. 2006). Lastly, Tang et al. found that PCT had a low diagnostic performance in differentiating sepsis from SIRS in critically ill adult patients, with an AUC of 0.79, a positive likelihood ratio 3.03, and a negative likelihood ratio 0.43 (Tang et al. 2007).

Actually, we need to keep in mind that PCT is potentially a biomarker of a state or syndrome (SIRS/sepsis/severe sepsis) not an indicator of a disease (unlike BNP which is a biomarker of a disease: CHF). A recent study nicely summarized the complexity of the evaluation and potential usefulness of PCT. Gaini et al. included 194 patients admitted in an internal medicine department; with suspected community-acquired infections and sepsis (Gaini et al. 2006); 106 had either infection or sepsis. They compared accuracy of Lipopolysaccharide-binding protein (LBP, an acute-phase protein that has been suggested as a marker of infection, because this protein has a role in the innate immune response in binding to lipo-polysaccharide), PCT, CRP, and IL-6 (which has a central role in inducing the synthesis of acute-phase proteins such as CRP, PCT and LBP). In a ROC curve analysis to distinguish between non-infected patients and infected patients, CRP and IL-6 had the highest AUC values of 0.83 and 0.82, compared to 0.77 for PCT. In a ROC analysis to distinguish between patients with noninfectious SIRS and patients with sepsis/severe sepsis, IL-6, LBP and CRP had an AUC of 0.87, 0.86 and 0.84, respectively; compared to 0.75 for PCT. However, in a ROC analysis to distinguish between patients with sepsis and patients with severe sepsis, PCT performed best with an AUC of 0.74. We could explain these conflicting results by the higher accuracy of PCT as a prognostic indicator but not as a diagnostic biomarker. As authors stated “CRP, IL-6 and LBP appear to be superior to PCT as diagnostic markers for infection and sepsis in patients. PCT appears to be superior as a severity marker”. This study also highlighted that biomark-ers examined could have different test qualities depending on the study population. Other reports suggested that PCT could have a prognosis value in ICU or ED (Hausfater et al. 2002). For example, Clec’h et al. included 75 patients with suspicion of sepsis in an ICU (Clec’h et al. 2006). A cutoff value of 6 ng/mL on day 1, separated patients who died from those who survived with 87.5% sensitivity and 45% specificity. CRP was not helpful for predicting mortality.

In emergency medicine medical literature is poorer than in critical care (Luzzani et al. 2003; Simon et al. 2004) and also leads to conflict results.

### Evaluation of procalcitonin in ED

Hausfater et al. prospectively evaluated serum PCT concentrations in patients who presented to an ED with suspected infectious or inflammatory disease (criteria of inclusion: order of CRP by EP). Of 195 study patients, 68 (30%) had final diagnosis of systemic infection, and 24 of those 68 had elevated serum PCT levels (>0.5 ng/mL). The PCT level had a sensitivity of 0.35 and specificity of 0.99 for the diagnosis of systemic infection. In multivariate analysis, the PCT level was the only independent variable associated with this diagnosis (OR 43.5; p = 0.0004); in contrast, the CRP level was not. The AUC of PCT for discrimination between patients with and without infection was 0.79 (Hausfater et al. 2002). Guven et al. included 34 patients with signs of SIRS and found that predictive accuracy for sepsis expressed as AUC was 0.88 for PCT, 0.44 for WBC, and 0.34 for CRP (p < 0.05) (Guven et al. 2002).

However, Chan et al. found that PCT was not a better marker of bacterial infection than CRP for adult ED patients, but was a useful marker of the severity of infection. Compared with CRP, PCT had a comparable sensitivity (69.5% versus 67.2%), a lower specificity (64.6% versus 93.9%), and a lower AUC (0.689 versus 0.879). However PCT levels, but not CRP levels, were significantly higher in bacteremic and septic shock patients. Multivariate logistic regression identified that a PCT level ≥2.6 ng/ml was independently associated with the development of septic shock (OR, 38.3) (Chan et al. 2004). Indino et al. also reported poor results with PCT (cutoff point of 0.5 ng/ml) for the diagnosis of a systemic infection in ED patients (on whom 57% had a final diagnosis of systemic infection), with a sensitivity of 0.57, a specificity of 0.85 (Indino et al. 2007).

In 208 elderly patients aged 75 and older admitted to an acute geriatric care unit, Strucker et al. found that PCT had good specificity (94%), but very low sensitivity (24%) in detecting infection (Stucker et al. 2005). One of the explanations is that they used a less sensitive test (Liaison BRAHMS PCT, limit of quantification 0.3 ng/mL). However, Caterino, also found that in elder ED patients, AUC for PCT for bacteremia was low as 0.70 (Caterino et al. 2004). Recently, Hausfater et al. included (Hausfater et al. 2007), in a prospective, monocentric non interventional study, 243 patients with body temperature ≥38.5 C. PCT assay with a 0.2 mg/L cutoff value had a sensitivity of 0.77 and a specificity of 0.59 for the diagnosis of bacterial/parasitic infection. Furthermore, 51% of the patients with PCT ≥5 mg/L had critical illness (death and/or ICU admission) in comparison with 13% of the patients with lower PCT values.

Because of the low sensitivity but excellent specificity and positive predictive value for this test, an elevated PCT concentration (higher than 0.5 ng/mL) indicates ongoing and potentially severe systemic infection with an increased risk of fatal outcome, whereas normal values do not exclude the possibility of an infectious process in its beginning stages (Fig. 5).

## Evaluation of PCT in specific diseases

### Accuracy of PCT to distinguish bacterial from aseptic meningitis

In patients with suspected meningitis and a positive Gram staining of the cerebrospinal fluid, the diagnosis of bacterial meningitis (BM) is proven. Further tests are not necessary, and empirical antibiotic treatment should be started immediately. However, in 30% to 40% of cases, the Gram-stained smear shows no bacteria. Thus, as recommended, patients in whom BM cannot be completely ruled out usually receive empirical antibiotic treatment until the microbiological findings or the clinical course excludes a bacterial origin. However, the cost of antibiotic therapy and its attendant hospitalization, as well as its potential adverse effects, have raised concern about giving unnecessary antibiotics in cases of nonbacterial meningitis (NBM). In adults, 4 studies evaluated PCT in meningitis. In 1999, Viallon et al. suggested that PCT had a higher diagnostic sensitivity (100%) and specificity (100%) than that of CRP to determine the BM origin of meningitis (Viallon et al. 1999). Jereb et al. evaluated the diagnostic performance of serum PCT levels in 45 adult patients (Jereb et al. 2001). A serum PCT level >0.5 ng/ml had a positive predictive value for BM of 100% and a negative predictive value of 93%. In 151 adults with confirmed meningitis admitted to our ED, we confirmed that PCT was a high predictor of BM (AUC of 0.99) compared to 0.75 for CRP ( p < 0.05), with a best threshold value of 2.13 ng/mL (Ray et al. 2007). Schwarz et al. included a total of 30 patients with a mean age of 52 yrs, having acute BM (n = 16) or NBM (n = 14) (Schwarz et al. 2000). PCT was the variable with the highest specificity for BM (100%), but there were false-negative findings in five patients with BM (a sensitivity of 69%). Persistently elevated or increasing PCT levels after 2 days were associated with an unfavorable clinical course. Recently, Viallon et al. confirmed that a significant and early decrease in serum PCT concentration was associated with favorable cure of BM (Viallon et al. 2005).

### PCT and community-acquired pneumonia

As much as 75% of all antibiotic doses are prescribed for acute respiratory tract infections (Prat et al. 2006). In a pilot study, Hedlund et al. (Hedlund and Hansson, 2000) suggested that PCT might aid the physician to differentiate typical bacterial community-acquired pneumoniae (CAP) etiology from atypical etiology: 8/9 patients with pneumonia caused by atypical agents had PCT levels <0.5 ng/ml compared with 6/27 patients with pneumonia caused by classic bacterial pathogens, mainly Streptococcus pneumoniae (p = 0.03). Mueller et al. included 545 patients with suspected lower respiratory tract infection, admitted to the ED (Muller et al. 2007). PCT had a higher diagnostic accuracy (AUC: 0.88) in differentiating CAP from other diagnoses, as compared to CRP (AUC: 0.76; p < 0.001) and total leukocyte count (AUC: 0.69; p < 0.001). PCT levels increased with increasing severity of CAP, classified according to the pneumonia severity index score (p < 0.001). To determine the prognostic role of PCT for severe CAP, 110 patients admitted in an ICU were prospectively studied. Boussekey et al. found that initial PCT level was significantly higher in patients who died during the ICU stay (5.6 ng/ml vs 1.5 ng/ml; p < 0.0001) (Boussekey et al. 2005). Christ-Cain and Mueller nicely performed three randomized studies to assess if a PCT-based therapeutic strategy (based on serum PCT concentrations as follows: antibiotic strongly discouraged when less than 0.1 ng/mL; discouraged when less than 0.25 ng/mL; encouraged when greater than 0.25 ng/mL; strongly encouraged when greater than 0.5 ng/mL) could reduce antibiotic use in lower respiratory tract infections (Christ-Crain et al. 2004). The use of PCT reduced the antibiotic use in lower respiratory tract infections/CAP and COPD exacerbation (Christ-Crain et al. 2006; Stolz et al. 2007). For example, in a randomized intervention trial, 302 consecutive patients with suspected CAP were included. PCT guidance reduced total antibiotic exposure (relative risk, 0.52; p < 0.001), antibiotic prescriptions on admission (85% vs. 99%; p < 0.001), and antibiotic treatment duration (median, 5 vs. 12 d; p < 0.001) compared with patients without PCT (Christ-Crain et al. 2006). Stolz et al. evaluated the efficacy and safety of PCT guidance compared to standard therapy with antibiotic prescriptions in patients experiencing exacerbations of COPD. PCT guidance reduced antibiotic prescription (40% vs. 72%, respectively; p < 0.0001) and antibiotic exposure (relative risk, 0.56; p < 0.0001) compared to standard therapy (Christ-Crain et al. 2006; Stolz et al. 2007).

### Role of PCT in various infectious diseases in adults

Viallon et al. demonstrated that serum PCT level may become a useful marker for the diagnosis of spontaneous bacterial peritonitis in 61 cirrhotic patients (cut-off value of 0.75 ng/ml, with a sensitivity of 95%, a specificity of 98%) (Viallon et al. 2000). However, Spahr et al. did not confirm this encouraging result (Spahr et al. 2001). Lemiale et al. found that a single PCT level was a poor predictor of 28-d adverse medical outcomes in women with pyelonephritis treated in the ED (Lemiale et al. 2007). Whether, PCT could be used and has the same diagnostic value in neutropenic and immunosupprssed febrile adult patients was suggested, but needs confirmation (Bernard et al. 1998; Ortega et al. 2004; Hausfater et al. 2007; Massaro et al. 2007).

### Discussion about conflicting results on procalcitonin

Differences in results in adult medical literature could be explain by: criteria of inclusion (fever, sepsis, SIRS…), population studied and prevalence of sepsis (ED, ICU, elderly patients), sample of patients, and primary end-points (accuracy to diagnose bacterial sepsis from inflammation or non non bacterial disease, differentiation between SIRS from septic shock, accuracy to predict death…), threshold values, methods of assay (use of an ultrasensitive assay (sensitivity of 0.06 ng/mL) PCT Kryptor® BRAHMS or PCT BioMerieux® vs LUMITEST® BRAHMS a relative insensitive assay vs a semi-quantitative assay PCT-Q® BRAHMS….).

However, the next important steps in assessing PCT diagnostic values should be to test the usefulness of PCT on the outcome (antibiotic administration, morbidity, mortality) in a randomized study as recently performed (Christ-Crain et al. 2004).

## Perspective in Biomarkers

It is beyond this article to describe all the new markers in cardiovascular diseases and/or in infectious diseases. However, readers should know that new biomarkers are currently evaluated (ST2 for prognostication in CHF patients, blood levels of endotoxin to predict adverse outcomes in ICU, diagnosis of viral infection by interferon-alpha levels, triggering receptor expressed on myeloid cells-1-TREM- as reliable marker of infection during severe sepsis and pneumonia…) (Januzzi et al. 2007). Finally, other studies suggested that instead of a single marker, a combination of biomarkers may improve diagnosis of infection (Kofoed et al. 2007).

## Conclusion

There is evidence that BNP and NT-proBNP are reliably markers of CHF in acute dyspnea. Their prognostic values are strongly suggested. Used in conjunction with other clinical information, rapid measurement of BNP or NT-proBNP reduced time to discharge and total treatment cost of patients. Overall accuracy of PCT is higher than that of CRP to differentiate bacterial infections from non-bacterial infections. In suspected lower tract infections (CAP or COPD exacerbation), PCT guidance reduced total antibiotic exposure and antibiotic prescriptions on admission. PCT and B natriuretic peptides should be promoted in EDs to aid triage, and improve outcomes.

## Figures and Tables

**Figure 6:**
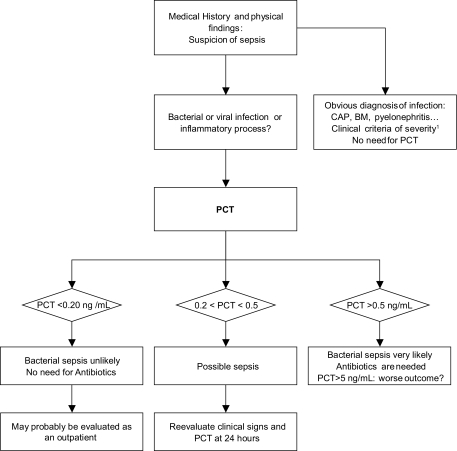
Suggested diagnostic strategy based on PCT levels in EDs. ^1^Systolic Blood Pressure less than 90 mmHg, mottling, oliguria, lactate >2 mmol/L or other signs of septic shock or trouble CSF after lumbar puncture, obvious clinical presentation of community-acquired pneumoniae (thoracic symptoms, fever, and consolidation on chest x-ray).
